# Relative Biological Effectiveness—Clinical Practice at US Proton Therapy Centers

**DOI:** 10.1016/j.ijpt.2025.101212

**Published:** 2025-11-14

**Authors:** Armin Lühr, Radhe Mohan, Anita Mahajan, Helen A. Shih, Harald Paganetti

**Affiliations:** 1 Department of Physics, TU Dortmund University, Dortmund, Germany; 2 Department of Radiation Physics, The University of Texas MD Anderson Cancer Center, Houston, TX, USA; 3 Department of Radiation Oncology, Mayo Clinic, Rochester, MN, USA; 4 Department of Radiation Oncology, Mass General Brigham and Harvard Medical School, Boston, MA, USA

**Keywords:** Relative biological effectiveness, Proton therapy, Linear energy transfer

## Abstract

**Purpose:**

Proton therapy, with its high conformality and superior dose distribution, is becoming an increasingly prevalent modality in radiation oncology. Although a generic relative biological effectiveness (RBE) value of 1.1 for treatment planning has been adopted, evidence suggests that RBE varies with dose, linear energy transfer (LET), and biological endpoints. This variation raises concerns about underestimating the RBE-weighted dose, which may significantly impact treatment outcomes. To assess the awareness and management of RBE variability in clinical practice, we conducted a survey among US proton therapy centers.

**Materials and Methods:**

The survey consisted of 32 questions grouped into subtopics, such as awareness of RBE variability, practices to account for variability, and future needs. It was distributed among 29 centers through the auspices of the Particle Therapy Co-Operative Group – North America.

**Results:**

The response rate was 80% and revealed significant apprehension about the assumption of a constant RBE. All respondents used a fixed RBE of 1.1, yet expressed varying degrees of concern about its appropriateness. Most centers report unanticipated toxicities and local recurrences potentially linked to an underestimation of RBE, highlighting the need for revisiting current practices. The survey indicated that nearly all centers consider RBE variability during beam arrangement, and some perform LET and variable RBE calculations for patient-specific treatments. There is a strong consensus on the need for multi-institutional databases, studies to accumulate clinical evidence, and education on proton RBE to better understand RBE's clinical implications.

**Conclusions:**

This study is an overview of current clinical practices regarding proton RBE and identifies key priorities for future research. Although deviations from the generic RBE of 1.1 is commonly considered in treatment planning, the absence of standardized approaches leads to inconsistencies. Developing consensus guidelines and integrating advanced RBE and LET-based models into treatment planning could improve the precision and safety of proton therapy.

## Introduction

Proton therapy is emerging as an increasingly accessible, highly conformal treatment option in radiation oncology. Protons possess a higher biological effectiveness compared to conventionally used photons, a factor that must be considered in patient treatment. Currently, a generic relative biological effectiveness (RBE) value of 1.1 is applied for both tumors and normal tissues,^1^, ^2^ despite clear evidence indicating that RBE is variable. Radiobiological experiments have demonstrated that RBE varies as a function of dose, linear energy transfer (LET), and biological endpoints.^2^, ^3^, ^4^, ^5^, ^6^

Given the rapidly increasing number of patients being treated with protons, more and more data on treatment outcomes are becoming available. The lower integral dose of proton therapy compared to photon radiation techniques would suggest a lower incidence of normal tissue toxicities. However, an underestimation of RBE might lead to unexpected side effects, particularly in distal portions of beams where LET is high. The LET effects may be more pronounced in late-responding normal tissues with a low α/β.^2^, ^7^

RBE variations are of particular concern in normal tissues, as the commonly used generic RBE value of 1.1 was originally defined conservatively based on tumor control considerations.^8^ Clinical evidence for RBE effects in patients has been inconsistent—some studies report significant deviations from 1.1,^9^, ^10^, ^11^, ^12^ whereas others support the validity of the 1.1 assumption.^13^, ^14^, ^15^ Beyond variations in the overall RBE, some data suggest differences in underlying biological mechanisms.^13^, ^14^, ^16^

Most RBE-related clinical studies have focused on central nervous system and head and neck toxicities. In some cases, incorporating LET in addition to dose improved correlation with observed effects.^17^, ^18^ For example, LET was associated with brain necrosis risk in only a subset of adult brain and head and neck patients.^19^ Reported rates of brain and brainstem necrosis after proton therapy vary considerably.^20^, ^21^, ^22^, ^23^, ^24^, ^25^, ^26^, ^27^, ^28^

These findings have sparked controversy and raised concerns about the current use of a fixed RBE. A recent position paper from the NRG Oncology Particle Therapy Work Group reviewed proton RBE and issued recommendations for future proton therapy trials.^29^ It acknowledges that, in the future, RBE values used for treatment planning and outcome assessment would need to be variable and different from the current standard. The report also identifies incorporating both dose and LET into planning as a promising intermediate step toward the ultimate goal of case-specific variable RBE-based treatment planning.

In clinical practice, most proton therapy centers still apply the standard RBE of 1.1 but implement additional measures to mitigate uncertainty.^30^ A prior survey of European proton centers^31^ found that while all assumed a constant RBE of 1.1, many adjusted treatment margins or field arrangements to account for possible RBE variability. Other measures—such as calculating LET and RBE during routine planning—were rarely used at the time due to the lack of standardized clinical tools. Recently, individual treatment planning systems (TPS) providers have made LET-based optimization commercially available, which, on one hand is a step forward, but on the other may lead to greater variability in how centers address potential RBE effects. Inconsistent approaches to RBE variation will complicate cross-institutional comparisons of clinical outcomes.^32^

A key question is whether practitioners at proton therapy centers find it necessary to revise current clinical practice. As a first step toward enhancing future clinical care, it is crucial to gain a comprehensive understanding of the current practice. This will help identify existing knowledge gaps, clinical needs, and areas for further research related to proton RBE. Accordingly, the purpose of this study is to assess the awareness and concerns among American proton therapy centers regarding the variation of proton RBE, its impact on clinical practice and outcomes, and the future needs to account for RBE variations based on a survey.

## Materials and methods

This survey was designed as an online questionnaire using Jotform (Jotform, San Francisco, USA). It consists of 32 questions in total. The actual number of questions each participant was queried ranged from 14 to 32, with some questions conditional upon previous responses. The questions were organized into 8 sections as follows:

• Demographic information about the respondents.
• Awareness of the variability of proton RBE.
• Proton beam arrangement practice.
• Accounting for RBE variability in treatment planning.
• Prescription of variable RBE.
• Calculation of LET and RBE.
• Consideration of RBE in clinical follow-up.
• Future needs.

A complete list of questions in the questionnaire can be found in the Supplementary Materials.

The questionnaire was distributed in a 2-step process to improve the consistency and comparability of the responses. In the first step, all proton therapy centers in the US that were on the Particle Therapy Co-Operative Group’s (PTCOG) list of Facilities in Operation^33^ website (41 centers) were asked to provide contact details, including email addresses of the most appropriate persons (up to 2 individuals) who would be responsible for completing the survey as representatives of their center. A total of 29 centers provided the required contact information, while 12 centers did not respond. In the second step, an invitation email with a link to the online questionnaire was sent by the PTCOG North America (PTCOG-NA) to the aforementioned representatives of the centers. It was requested that responses be limited to 1 representative radiation oncologist and one clinical medical physicist of the centers. Respondents were encouraged to consult their internal colleagues when answering the survey. The online survey was closed as of January 1st 2024.

The responses were downloaded by PTCOG-NA. The name and city of the proton centers were used to determine the number of responding centers and responses per center. Subsequently, the survey was analyzed in an anonymous manner. For this purpose, information on the proton centers was separated from the responses by PTCOG-NA, and the blinded data were forwarded to the authors for further analysis. The chi-square and t-test were performed to determine statistical significance, and a *P* value below .05 was considered statistically significant.

## Results

### Demographics

Of the 29 centers that received a link to the questionnaire, a total of 23 centers (80%) responded. Based on PTCOG’s list of Facilities in Operation in 2024, these centers had been in clinical operation for an average of approximately 10 years, which compared well with the average operating time of about 9 years for all 48 US proton facilities.

A total of 27 responses were recorded, and the raw data for all responses are provided as de-identified Supplemental Data. Of the 27 respondents, 6 described their role in proton therapy as treating physician (22%) and 21 as clinical physicist (78%) [Question (Q)1]. Three centers were represented by responses from both a treating physician and a clinical physicist, 16 centers by a clinical physicist, 3 by a treating physician, and for 1 center, 2 respondents indicated to be clinical physicists. All quantitative results presented below represent the absolute or relative frequency of responses provided for the survey questions.

### Concerns about the assumption of a constant RBE of 1.1

**Figure 1:**
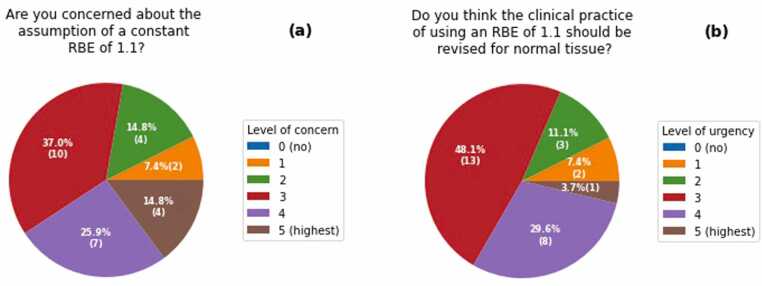
Assumption of a constant relative biological effectiveness (RBE) of 1.1: Responses on the level of (a) overall concern [Q3] and (b) urgency for revision of current practice for normal tissue [Q4]. Distribution in percent (number of responses).

All participating centers considered the dose weighted with a fixed RBE of 1.1 for prescription doses and constraints [Q14, Q15]. At the same time, all centers were, to some degree, concerned about the assumption of a constant RBE of 1.1 [Q3] (Figure 1). On a scale of 0 (lowest, none) to 5 (highest), only a few respondents (7% of responses) rated their level of concern as low (levels 0 and 1), while the majority (52% and 41%, respectively) said they were either concerned (2 and 3) or very concerned (4 and 5). Radiation oncologists seemed to be concerned to a higher level than medical physicists (Supplement Figure S1). However, none of the differences in the responses between radiation oncologists and clinical physicists in this section reached statistical significance given the small number of radiation oncologists.

A majority of responders (59%) indicated that they have observed unanticipated toxicities or local tumor recurrences at their institution that they suspect to be ascribable to the assumption of an RBE of 1.1 [Q2]. This view was shared by two-thirds of the medical physicists but only one-third of the radiation oncologists (Supplement Figure S2). Nearly all (88%) of those who observed such unanticipated toxicities or local tumor recurrences expressed a high level of concern (3-5) about using an RBE of 1.1. Most centers saw the need to review the current clinical practice, albeit with varying levels of urgency. For normal tissues, the urgency of reconsidering the handling of RBE was on average rated high [Q4] (Figure 1). For tumors, also a majority of centers saw the need for revision [Q5], though with lower urgency than for normal tissue (Supplement Figures S3 and S4).

### Consideration of RBE variability in beam arrangement and in treatment planning

**Figure 2:**
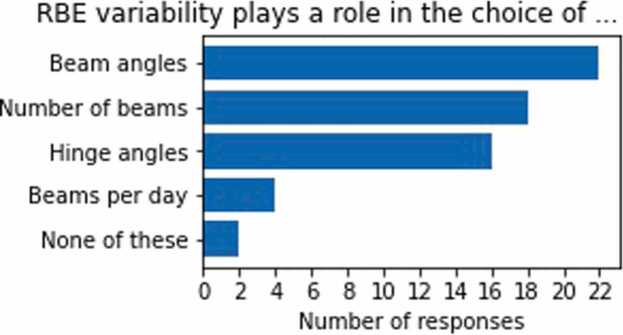
Proton beam arrangements that are influenced due to relative biological effectiveness (RBE) variability [Q9] as stated by the 26 responders who avoid beams stopping near organs at risk (cf. [Q6]). In total, 62 responses.

While none of the centers prescribed doses other than using the dose weighted by a fixed RBE of 1.1, nearly all respondents (93%) took into account that the RBE is variable when selecting proton beam arrangements [Q9]. RBE variability frequently played a role in the choice of beam angles (81%), number of beams (67%), and hinge angles between the beams (59%, Figure 2). The restrictions on the (minimum) hinge angle between proton beams were applied by about half (48%) of the respondents to most or all treatment sites and by about the other half (48%) to only some treatment sites [Q8]. All but one respondent (96%) stated that they avoid beam arrangements in which a beam stops in front of or inside an organ at risk (OAR) [Q6] (Supplement Figure S5). This was most commonly achieved by, for example, avoiding beam angles that result in beams stopping in or close to an OAR (85%), by assigning low weights to beams stopping in or close to an OAR (70%) or, less frequently, by extending beam range to place the end of range beyond an OAR ("shoot-through") (11%) [Q7].

**Figure 3:**
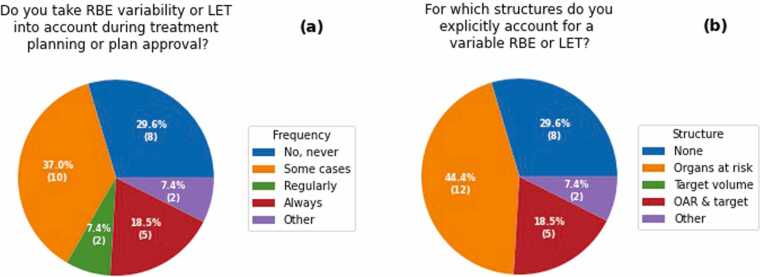
Taking variable relative biological effectiveness (RBE) or linear energy transfer (LET) into account during treatment planning or plan approval: Responses on (a) the frequency of their consideration [Q10] and (b) the considered structures [Q11]. Distribution in percent (number of responses). OAR, organ at risk.

**Figure 4:**
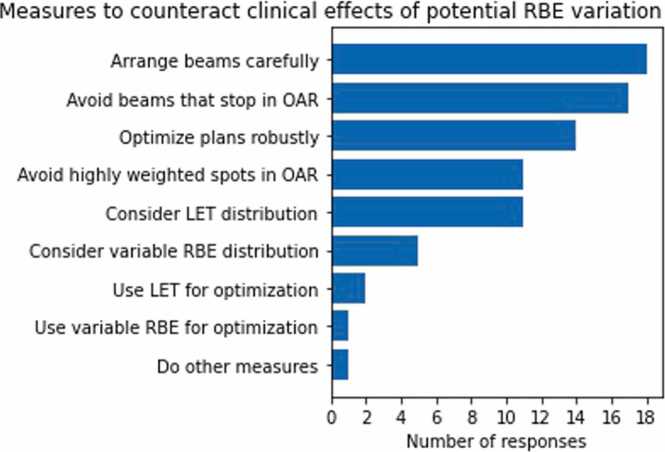
Measures applied to counteract clinical effects of a variable relative biological effectiveness (RBE) [Q12] by the 19 respondents who take at least in some cases variable RBE or linear energy transfer (LET) into account (cf. [Q10]). In total 80 responses. OAR, organ at risk.

The variability of RBE or LET was considered during various stages of treatment planning or for plan approval by 19 responders (63%) [Q10], either in selected cases (37%), regularly (7%), or even always (19%) (Figure 3). All centers that explicitly considered a variable RBE or LET did so for OARs, but some centers (19% of responses) did it for tumors also [Q11] (Figure 3). Nearly all of these (18 out of the 19 responses) applied at least 3 different measures to counteract potential clinical effects of RBE variation [Q12] (Figure 4). The most frequent measures were carefully considering beam arrangements (18); avoiding proton beams that stop in or adjacent to OARs (17); performing robust optimization for range and positioning uncertainties, thereby smearing high LET values (14); avoiding highly weighted spots close to critical structures (11); and considering the LET distribution in the evaluation of treatment plans (11). In contrast, some measures were only rarely applied, such as using a variable RBE model in treatment plan optimization (1); incorporating LET in treatment plan optimization (2); or using a variable RBE model in the evaluation of the treatment plan (5). Plans that were designed to take variable RBE or LET into account were typically evaluated, first, conventionally on a dose distribution weighted with a fixed RBE = 1.1 and, second, additionally on the LET distribution (14) or a dose weighted with a variable RBE (5) [Q13].

### LET and variable RBE calculations

About half, that is, 15 responders (56%), reported performing patient-specific LET or variable RBE calculations, including 10 that stated that they use them in some way to treat patients in clinical practice [Q16] (Supplement Figure S6). Brain (13), head and neck (11) and base of skull (8) were the most frequent tumor sites where institutions perform patient-specific LET or variable RBE calculations, followed by breast and prostate (both 4) [Q19]. Most of these institutions (11 responses) performed these calculations only occasionally (in less than or equal to 1 out of 10 patients), whereas only one did so frequently (more than 1 out of 10 patients), and 3 responders always did this [Q18] (Supplement Figure S7). Different (combinations of) software and systems were being used to perform the required calculations [Q21, Q22]. This included commercial software such as the TPS RayStation (10), Eclipse (3) and Monaco (1). However, only eight responders exclusively used a TPS for this purpose. The remaining 7 reported that they utilized in-house software, either exclusively (2) or in combination with a clinical TPS (5). MCsquare was the most frequently mentioned Monte Carlo software (3).

Patient-specific LET or variable RBE was integrated into clinical practice in various ways, such as in treatment planning, plan evaluation or approval (10), retrospective analysis for patient follow-up (10), addressing unexpected toxicity or recurrence (8), and for clinical research purposes (7) [Q17]. In the context of clinical decision-making, it was reported that distributions of LET (10), variable RBE-weighted dose (5), and variable RBE (4) are calculated as quantities related to RBE [Q20]. Notably, all centers using these calculations for retrospective analyses of individual patients had observed unexpected events attributed to the assumption of an RBE of 1.1. Only a few centers (3 responses) attempted to fit RBE model parameters to clinical data from such retrospective studies.

### Future needs

Moving forward, there was a strong desire across all centers to better understand RBE. Most respondents felt the need for more education regarding clinical use of proton RBE (89%) [Q26]. To this end, they considered educational sessions at conferences (67%), dedicated teaching articles or reviews (56%), webinars (51%), and topic-specific workshops (48%) as relevant and suitable formats [Q27].

On the other hand, more clinical evidence and empirical data were required to improve the understanding of RBE. All but 1 center (96% of responders) saw the need for a multi-institutional database of patient outcomes focusing on the question of proton RBE [Q32] (Supplement Figure S8). Furthermore, a majority of respondents (74%) thought there is a need for more preclinical experiments to understand clinical proton RBE [Q30]. While they all advocated in vivo experiments for this purpose (Supplement Figure S9), a few (26%, mostly radiation oncologists) also saw a benefit in further in vitro cell experiments [Q28].

## Discussion

This study demonstrates that RBE has manifested as a clinically relevant issue known to practitioners at proton therapy centers in the United States. A majority of centers have observed toxicities they suspect to be linked to RBE, and nearly all are concerned about an inappropriate assumption of a fixed RBE of 1.1. While all centers participating in the study have been using a fixed RBE of 1.1 for clinical treatment, most see the need to review the current clinical practice of using this simplified RBE concept. The highest urgency to consider a variable RBE for proton treatments has been expressed for normal tissue, particularly in the brain, head and neck, and base of skull region, which seems reasonable both from a clinical and physical point of view.^5^, ^6^, ^30^ Firstly, much of the clinical evidence for a variable RBE effect to date comes from normal tissue effects in these treatment sites.^34^ Secondly, proton irradiation in entities with relatively homogeneous tissue, little anatomical variation, and short proton ranges can lead to steep dose and LET gradients. This can result in increased variable RBE in the vicinity of the tumor (ie, clinical target volume) where, typically, serially organized OARs with low α/β values (eg, parts of the optical apparatus) can be located. In tumors, on the other hand, the clear demonstration of a clinical effect attributable to RBE variability is still lacking. In clinical data, the sensitivity to radiation can vary both within a tumor and from patient to patient, and a locally limited RBE increase seems to be more difficult to detect than for normal tissue. In line with this, the proton centers see less urgency to revise the current RBE concept for tumors than for normal tissue, maybe also because a constant RBE of 1.1 is considered as a conservative choice for tumor control, except for some specific tumors with very high α/β values. An increase in biological effect within solid tumors may conveniently increase tumor control without negatively impacting side effect risks. Even though many survey respondents see a lower urgency for tumors to revise the RBE concept, they still express uncertainty and concern regarding the RBE of protons for tumors.

The current generally adopted clinical strategy in the US to counteract the potential adverse effects of a variable RBE through considerations during treatment planning, for example, by carefully selecting the beam arrangements, is in line with the strategies implemented at all European proton therapy centers.^31^ It is possible that the widespread implementation of these countermeasures is indeed successful and contributes to the fact that the published incidence of adverse events attributable to a variable RBE is lower than would be expected based on simple in vitro-based RBE modeling.^30^

With the difficulty of defining reliable RBE values for normal tissue complications, a safe strategy adopted by few centers is to maintain current dose constraints but reduce an estimated biological effectiveness in regions of interest via, for example, LET-based plan optimization or other RBE-related quantities such as dirty dose.^6^, ^35^, ^36^, ^37^ About half of the reporting American centers performed patient-specific LET or variable RBE calculations. About one-third of the centers used results from LET calculations to support patient treatment while European centers nearly never did so (also because tools to calculate RBE-related quantities in a clinical setting were missing at the time of the European study). The latter rather used such calculations, if available, for retrospective analyses and research.^31^

The availability of empirical RBE models based on clinical data would be an attractive step towards revising the clinical practice of a constant RBE of 1.1. In a few selected cases, for example, for the brain, first empirical models have been suggested^11^, ^12^ and recently confirmed in an independent validated study.^18^ Because of potential discrepancies between clonogenic endpoints and clinical outcomes, LET-based phenomenological proton RBE models are mostly used qualitatively. These models can inform retrospective outcome analyses and indicate potential RBE-related trends but should not be used quantitatively for treatment optimization, as clinical α/β ratios differ from those derived from cell survival data. The rationale for LET-based rather than RBE-based optimization is that LET, as a physical metric, is not subject to the biological uncertainties inherent to RBE. However, LET optimization still requires a model to balance LET against dose, since elevated LET may not be biologically relevant in low-dose regions. Unlike comprehensive RBE modeling, LET-based optimization excludes tissue-specific radiobiological parameters such as α/β.

Currently, LET-based efforts are not standardized between centers, with a considerable variance concerning applied software, frequency, and reason to perform such calculations. Different definitions and scoring methods for LET can lead to substantially different LET-weighted doses and LET-based model estimates of RBE.^35^, ^38^ LET depends on calculation details, including averaging method (track- vs. dose-averaged), particle selection, scoring approach, and physical modeling parameters. These choices can produce substantial variations in LET-weighted doses.^39^ Harmonizing the reported LET among proton therapy centers is essential for consistent multi-center analyses.^29^, ^32^

More research as well as clinical and educational efforts seem necessary to guide practitioners on how to use available and upcoming technical tools to calculate RBE-related quantities most effectively and with confidence for improved patient outcome. A better understanding of RBE in a clinical setting is uniformly seen in this survey as a crucial factor for improved proton treatment in the future. Deducing RBE from clinical data remains challenging because the RBE effect has to be distinguished from dose–volume effects (eg, RBE should not be defined based on mean doses to a region). Voxel-based analysis may help to separate RBE and dose-volume effects. Furthermore, patient-specific risk and RBE modifiers need to be better understood.^40^ More efforts and a greater focus on conducting (quantitative) studies on clinical radiation effects of protons seem necessary, as very few centers in the US are currently working on researching relevant model parameters for biomathematical RBE models derived from patient outcome data.^41^ Clinical evidence and high-quality in vivo data, as well as targeted training of clinical staff in the clinical management of RBE, are essential. Radiotherapy societies such as PTCOG may play a crucial role in establishing relevant education opportunities as well as a multi-institutional patient outcomes database focused on the topic of proton RBE, which is generally agreed to be necessary.

This study has its limitations, as it only considers the clinical practice of proton therapy centers in the US, and not all centers shared their view. However, the experience of the participating centers, as measured in terms of their years of clinical operation, is comparable to the average operating time of all US proton centers. Also, only 3 centers provided input from both treating physicians and clinical physicists, while most centers (75%) were only represented by clinical physicists. Therefore, results could be biased toward opinions of physicists, and testing of statistical differences between responses of the 2 professions was limited due to the relatively small number of 6 treating physicians. No attempt was made to identify the 2 physicists belonging to 1 center in order to manually edit their responses during the analysis; however, this is expected to result in an error of less than 5%.

The survey reflects the current situation, and the responses may change over time, for example, with regard to LET and RBE calculations as clinical systems for LET simulation become more widespread in proton therapy centers. This study complements the results of 2 earlier studies focused exclusively on Europe^31^, ^32^ and together these provide the latest and most comprehensive overview of the clinical status of proton RBE considerations.

## Conclusions

This survey provides an overview on the current clinical practice with respect to uncertainties in proton RBE. A generic proton RBE of 1.1 is currently in use universally in proton therapy. Variations in RBE are being considered in mostly inconsistent manner, which impacts the ease of inter-institutional plan comparisons and outcome assessment. Considering the current practices in the use of proton RBE, the establishment of standardized guidelines for treatment planning, the accumulation of further clinical evidence, and dedicated education on proton RBE could improve the effectiveness and safety of proton therapy.

## Funding

This research did not receive any specific grant from funding agencies in the public, commercial, or not-for-profit sectors.

## CRediT authorship contribution statement

**Armin Lühr**: Conceptualization, Data curation, Investigation, Methodology, Visualization, Writing – original draft, Writing – review and editing. **Radhe Mohan**: Conceptualization, Investigation, Methodology, Writing – original draft, Writing – review and editing. **Anita Mahajan**: Resources, Writing – review and editing. **Helen A. Shih**: Methodology, Writing – review and editing. **Harald Paganetti**: Conceptualization, Investigation, Methodology, Writing – original draft, Writing – review and editing. All authors commented on the manuscript and have read and approved the final version.

## Declaration of Competing Interest

The authors declare that they have no known competing financial interests or personal relationships that could have appeared to influence the work reported in this paper.

## Data Availability

Research data are stored by PTCOG-NA and will be shared upon request.

## Footnotes

Appendix A Supplementary data associated with this article can be found in the online version at doi:10.1016/j.ijpt.2025.101212.
