# Supplementary material for: Relative Biological Effectiveness—Clinical Practice at US Proton Therapy Centers
Source: Int J Part Ther. 2025 Nov 14;18:101212. doi: 10.1016/j.ijpt.2025.101212 (PMC12686715; doi:10.1016/j.ijpt.2025.101212)
Supplement: Supplementary file 1 — Supplementary material [file mmc1.pdf]

Supplementary Material – Figures

Relative agreement to each of the answer options shown below the bars. Numbers above the bars show the relative number in % of the indicated group of respondents. The groups of respondents are color coded with **RED**: all; **BLUE**: Medical Physicists; **GREEN**: Radiation Oncologists

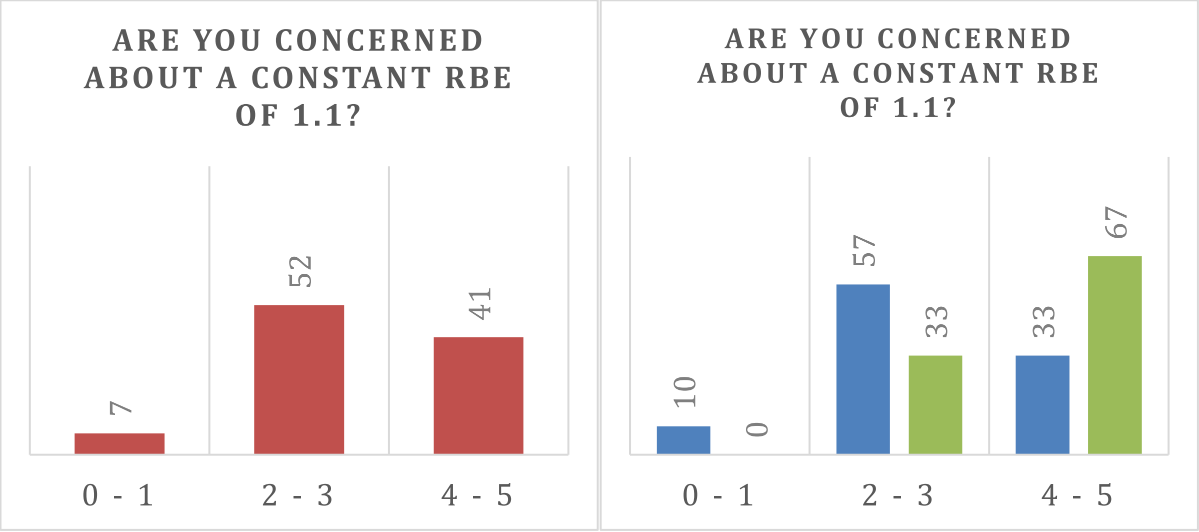

Figure S1: Are you concerned about the assumption of a constant RBE of 1.1? [Q3]. Level of concern from 0 (none) till 5 (highest).

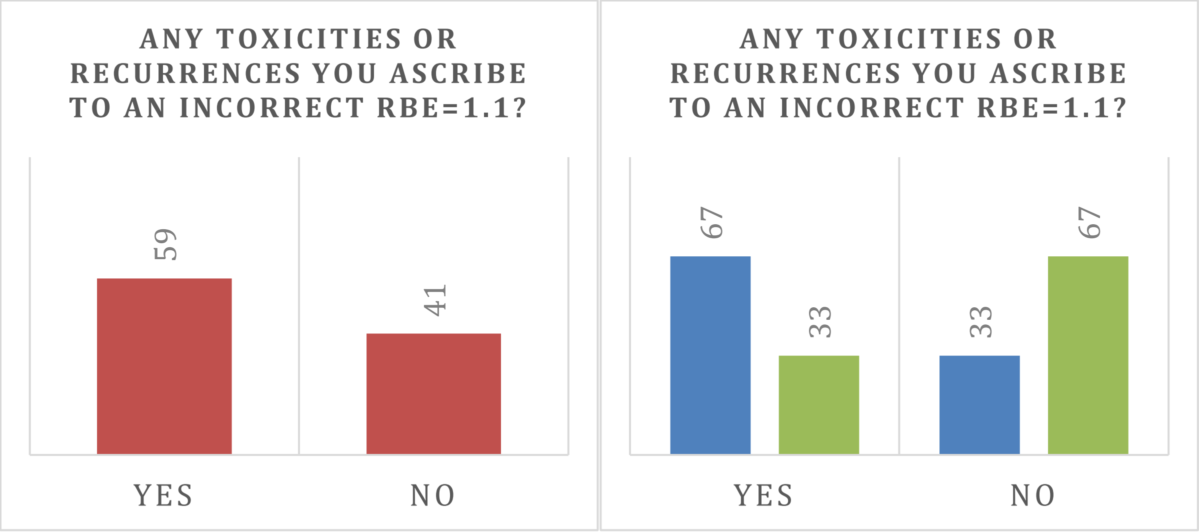

Figure S2: Have you observed any unanticipated toxicities or local tumor recurrences at your institution that you suspect may be ascribable to an incorrect assumption of RBE of 1.1? [Question (Q) 2]

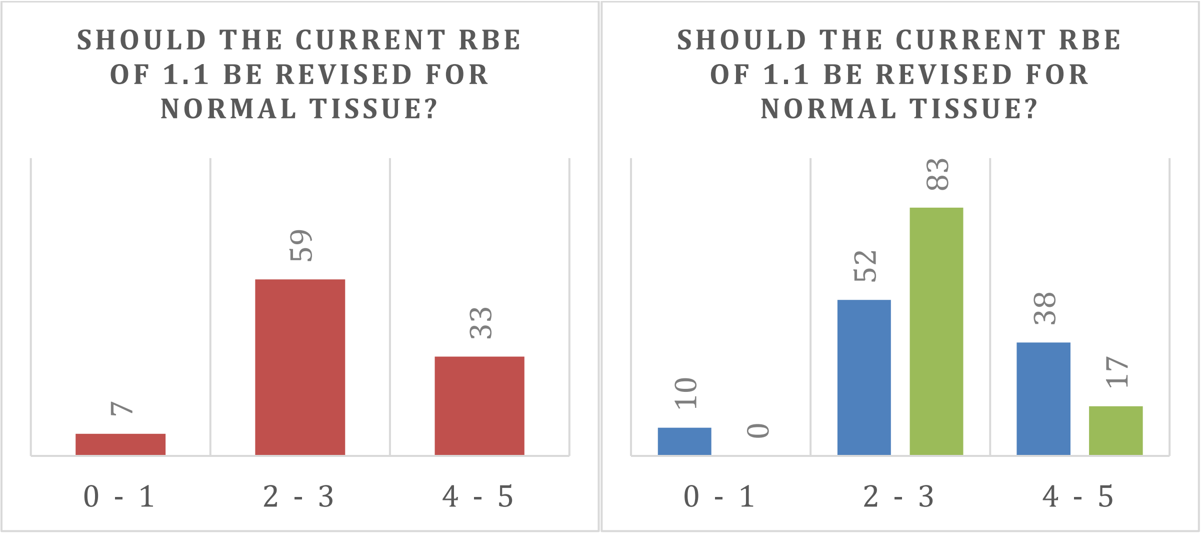

Figure S3: Do you think the current clinical practice of using an RBE of 1.1 should be revised for normal tissue? [Q4]. Level of urgency from 0 (none) till 5 (highest).

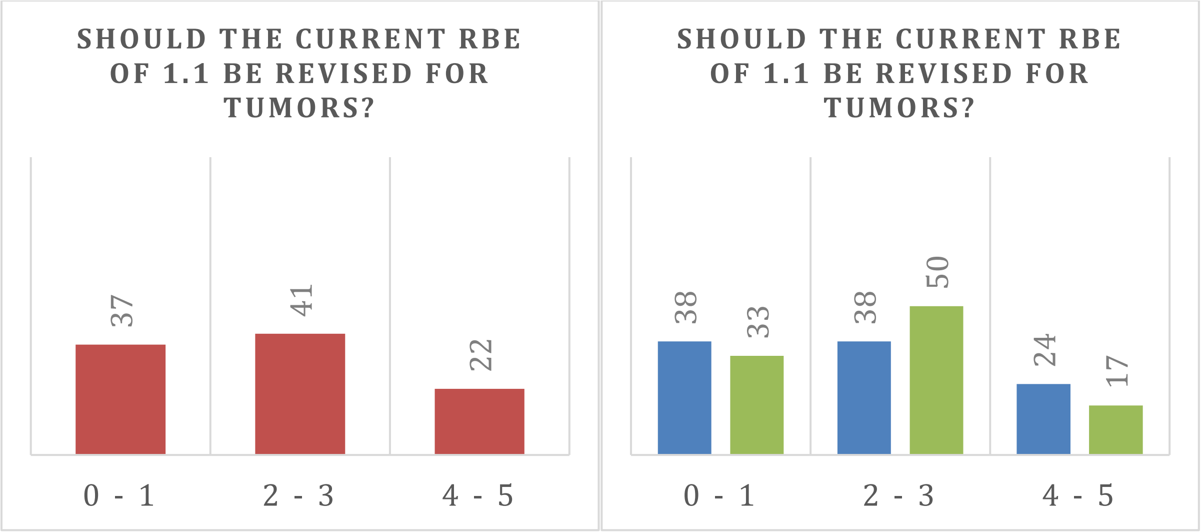

Figure S4: Do you think the current clinical practice of using an RBE of 1.1 should be revised for tumors? [Q5]. Urgency from 0 (none) till 5 (highest).

Prescription and treatment planning:

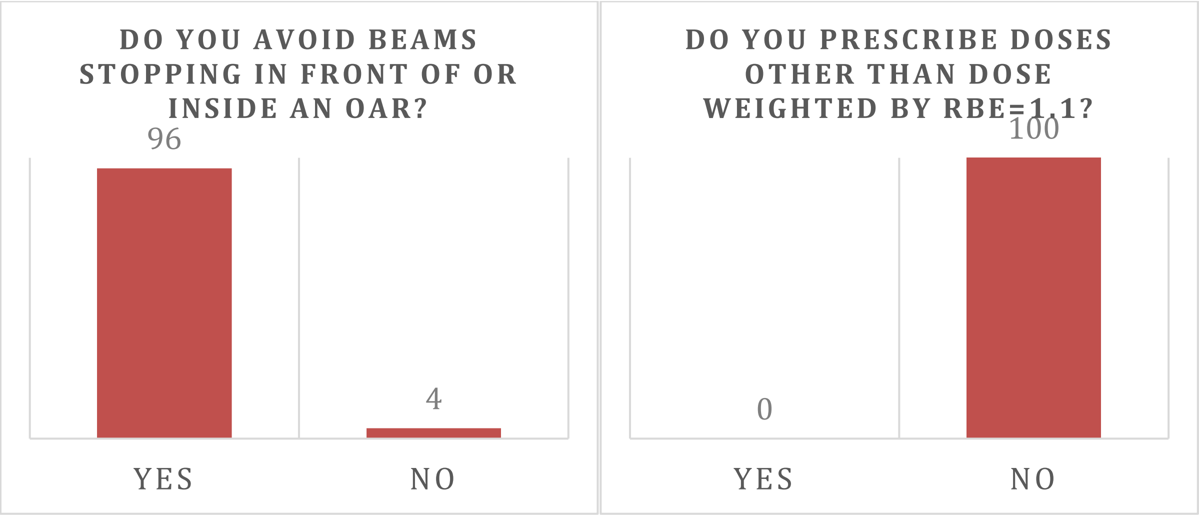

Figure S5: Left: Do you avoid beam configurations in which a beam stops in front of or inside an organ at risk (OAR)? [Q6]. Right: Do you prescribe doses and constraints for patient treatments other than using the dose weighted by a fixed RBE of 1.1? [Q14]

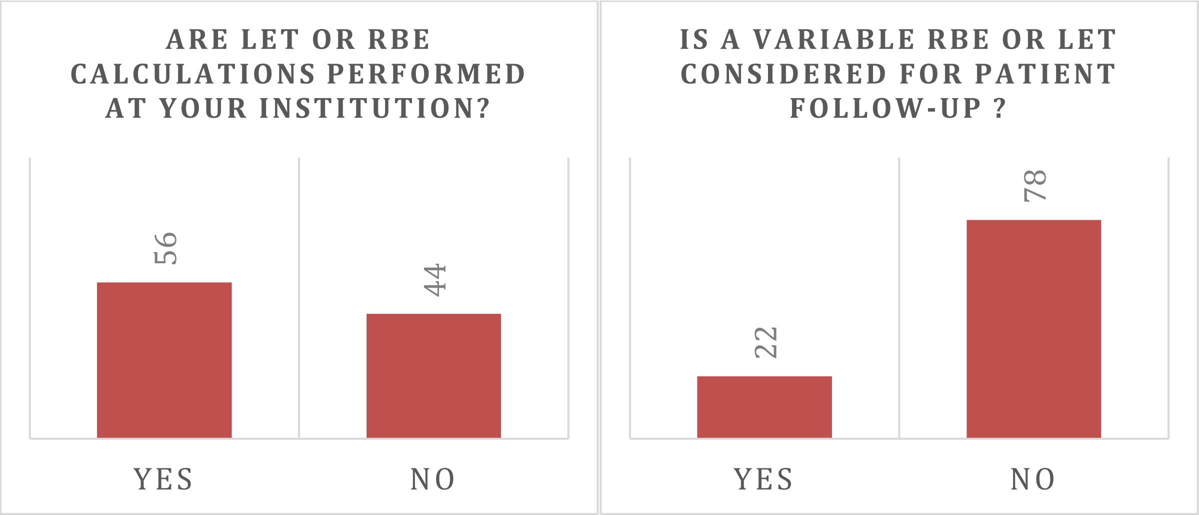

Figure S6: Left: Are patient specific LET or variable RBE calculations performed at your institution? [Q16]. Right: Is a variable RBE or LET considered for patient follow-up at your institution? [Q23]

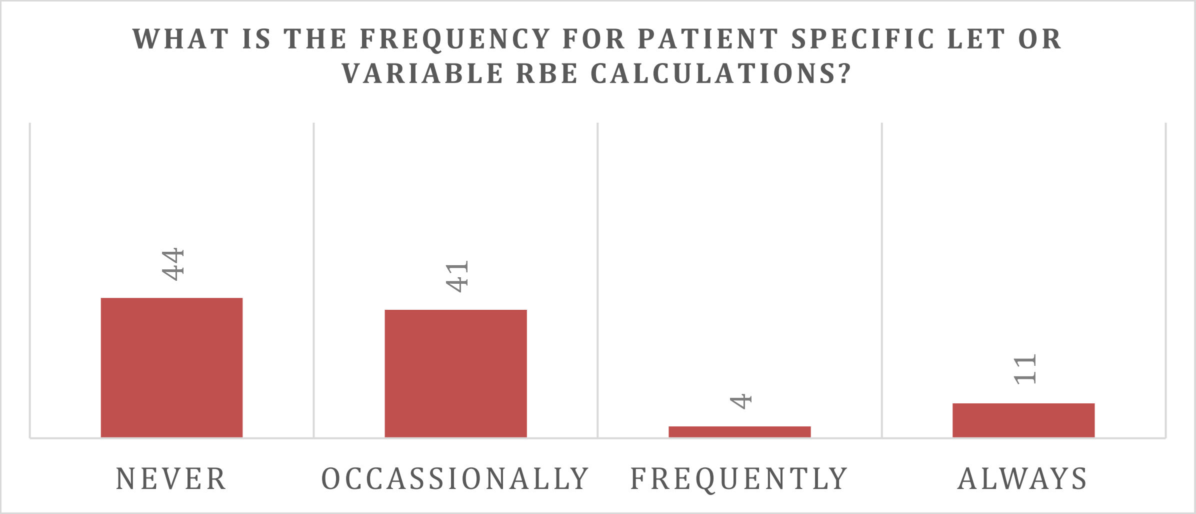

Figure S7: What is the frequency for performing patient specific LET or variable RBE calculations? [Q18].

Future needs:

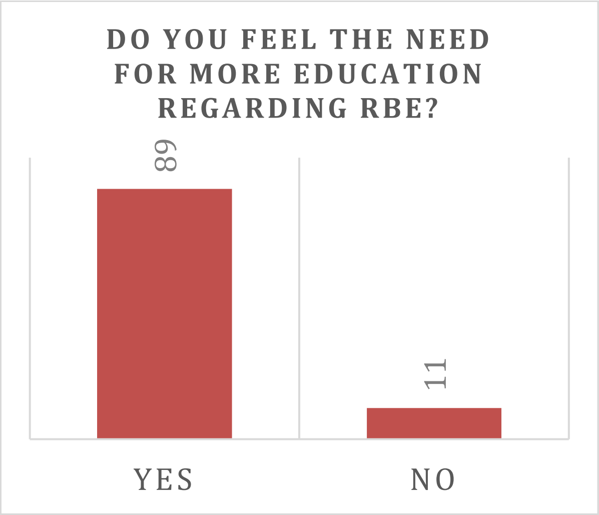

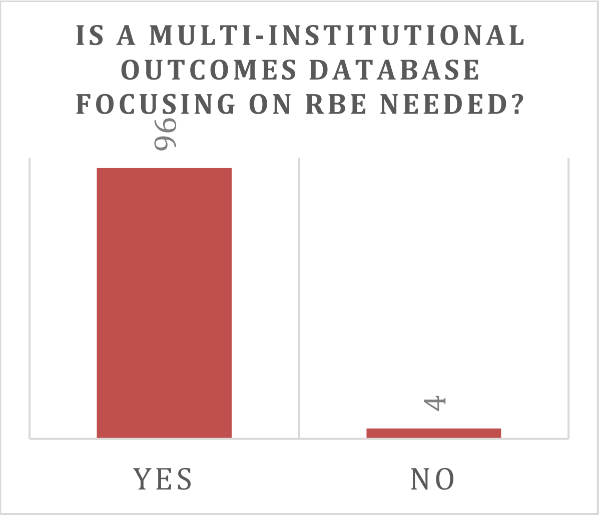

Figure S8: Left: Do you feel the need for more education regarding clinical proton RBE? [Q26]. Right: Do you think there is need for a multi-institutional database on patient outcomes focusing on the question of proton RBE? [Q32]

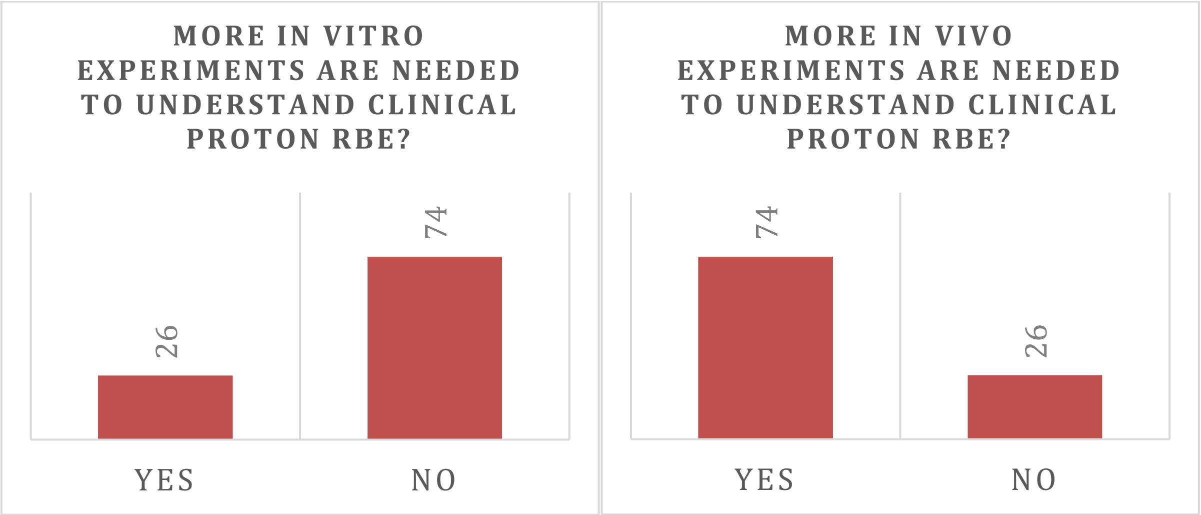

Figure S9: Left: Do you think there is a need for more in vitro experiments to understand clinical proton RBE? [Q28]. Right: Do you think there is a need for more in vivo pre-clinical experiments to understand clinical proton RBE? [Q30]
