# Supplementary material for: Relative Biological Effectiveness—Clinical Practice at US Proton Therapy Centers
Source: Int J Part Ther. 2025 Nov 14;18:101212. doi: 10.1016/j.ijpt.2025.101212 (PMC12686715; doi:10.1016/j.ijpt.2025.101212)
Supplement: Supplementary file 2 — Supplementary material [file mmc2.pdf]

Relative Biological Effectiveness (RBE) Questionnaire

Design: Armin Lühr, Radhe Mohan, Harald Paganetti

Introduction and Purpose:

Protons have a higher relative biological effectiveness (RBE) compared to conventionally used photons. Given the rapidly increasing number of patients being treated with protons, more and more data on treatment outcomes are becoming available. Some of these data have led to controversies and concerns regarding the average RBE of 1.1 used in current practice and the variability of RBE. Some centers have reported seemingly unexpected toxicities or treatment failures after proton therapy that they suspect may be due to the assumption of an RBE of 1.1.

There is currently no comprehensive understanding of the centers' views toward the following issues related to RBE:

- What are current practices for accounting for RBE at proton centers in the US?
- Are proton centers concerned about the assumption of constant RBE of 1.1?
- Do they think a revision of current clinical practice is warranted?

The purpose of this questionnaire is to assess among American proton therapy centers the awareness of variation of proton RBE, its impact on clinical outcomes and future needs to account for it in clinical practice with a focus on proton beam scanning. A similar survey was conducted previously among European centers, the research of which were published in https://doi.org/10.1016/j.radonc.2022.05.015.

This study will highlight current knowledge gaps, clinical needs and areas for further research related to clinical proton RBE to support improvement of clinical treatment in the coming years.

This survey is being sent to proton therapy centers in North America. We ask that responses be limited to one radiation oncologist and one physicist per center. Please feel free to consult your colleagues when responding to the survey.

Contents:

I.	Demographic information	3
II.	Awareness of variability of proton RBE	4
III.	Beam arrangements in consideration of high RBE at distal edges	5
IV.	Explicit accounting for variable proton RBE in treatment planning	6
V.	Prescription of a variable RBE in treatment planning	7
VI.	Patient specific LET or variable RBE calculations	8
VII.	Consideration of variable RBE or LET retrospectively	10
VIII.	Wish list of needs regarding proton RBE	11

I. Demographic information

The following information would be needed to resolve multiple replies from the same institution (it will be kept confidential).

- What is your institution's name?

- In which city and state is your institution located?

- -

1. What is your role in proton therapy?
    a. Treating physician
    b. Clinical physicist
    c. Research physicist
    d. Treatment planner
    e. Other (please specify)

II. Awareness of variability of proton RBE

2. Have you observed any unanticipated toxicities or local tumor recurrences at your institution that you suspect may be ascribable to an incorrect assumption of RBE of 1.1?
    a. Yes
    b. No
3. Are you concerned about the assumption of a constant RBE of 1.1?
    - Level of concern: 0 (lowest) to 5 (highest).
4. Do you think the current clinical practice of using an RBE of 1.1 should be revised for normal tissue?
    - Level of urgency: 0 (lowest) to 5 (highest).
5. Do you think the current clinical practice of using an RBE of 1.1 should be revised for tumors?
    - Level of urgency: 0 (lowest) to 5 (highest).

III. Beam arrangements in consideration of high RBE at distal edges

6. Do you avoid beam configurations in which a beam stops in front of or inside an organ at risk (OAR)?
    a. Yes
    b. No

CONDITIONAL:

IF YES: please respond to the following questions

ELSE: move on to topic IV

7. How do you avoid beams that may stop in or close to an OAR? [Select all applicable responses]
    a. Beam angles are avoided that result in beams stopping in or close to an OAR.
    b. Beams stopping in or close to an OAR are assigned low weights.
    c. Beam ranges are extended to place the end of range beyond an organ at risk ("shoot- through").
    d. Other (Please specify your strategy)
8. Do you apply restrictions on the (minimum) hinge angle between proton beams?
    a. No, never.
    b. For some treatment sites.
    c. For most treatment sites.
    d. Always.
9. Does the RBE variability play a role in the choice of … [Select applicable responses]
    a. The number of beams?
    b. The number of beams per day?
    c. The choice of beam angles?
    d. The hinge angles between the beams?

IV. Explicit accounting for variable proton RBE in treatment planning

10. Do you take into account RBE variability or linear energy transfer (LET) during treatment planning or plan approval?
    a. No, never.
    b. In some cases.
    c. Regularly.
    d. Always.
    e. Other (please specify)

CONDITIONAL:

IF A: move on to topic V

ELSE: please answer the following questions

11. For which structures do you explicitly account for a variable RBE or LET? [Select applicable responses]
    a. Organs at risk.
    b. Target volume.
    c. Other (please specify)
12. What measures do you apply to counteract potential clinical effects of RBE variation? [Select applicable responses]
    a. Carefully consider beam arrangements (e.g., additional beam or minimum hinge angle).
    b. Avoid proton beams that stop in or adjacent to organs at risk.
    c. Perform robust optimization for range and positioning uncertainties, thereby smearing high LET values.
    d. Optimize intensity modulated proton therapy (IMPT) to avoid highly weighted spots close to critical structures.
    e. Consider the LET distribution in the evaluation of the treatment plan.
    f. Consider a variable RBE model in the evaluation of the treatment plan.
    g. Use LET in treatment plan optimization.
    h. Use a variable RBE model in treatment plan optimization.
    i. Other measures (please specify)
13. How do you evaluate plans designed taking variable RBE or LET into account? [Select all applicable responses]
    a. Based on a dose distribution weighted with a fixed RBE=1.1 as well as LET distribution.
    b. Based only on variable RBE weighted dose distribution.
    c. Using variable RBE as well as RBE = 1.1 weighted dose to ensure traditional constraints are maintained in both cases.
    d. Other (please specify).

V. Prescription of a variable RBE in treatment planning

14. Do you prescribe doses and constraints for patient treatments other than using the dose weighted by a fixed RBE of 1.1?
    a. Yes
    b. No

CONDITIONAL:

IF YES: please answer the following questions

ELSE: move on to next topic VI

15. In which cases do you apply an RBE different from RBE = 1.1? [Select all applicable responses]
    a. Never.
    b. Individual clinician decision.
    c. For specific beam arrangements/treatment plans.
    d. For pediatric patients.
    e. For other specific cancer types (please specify).
    f. In clinical studies aiming at understanding the impact of a variable RBE.
    g. Always.
    h. Other (please specify)

VI. Patient specific LET or variable RBE calculations

16. Are patient specific LET or variable RBE calculations performed at your institution?
    a. Yes
    b. No

CONDITIONAL:

IF YES: please answer the following questions

ELSE: move on to next topic VII

17. How do results of LET or variable RBE calculations enter into clinical practice? [Select all applicable responses]
    a. Never used.
    b. During the treatment planning process.
    c. For plan evaluation or plan approval.
    d. In the course of robust optimization.
    e. For documentation.
    f. For retrospective analysis to support patient follow-up.
    g. When an unexpected toxicity or recurrence is observed.
    h. For clinical research purposes.
    i. Other (please specify)
18. What is the frequency of performing patient specific LET or variable RBE calculations?
    a. Never.
    b. Occasionally (less than or equal 1 out of 10 patients).
    c. Frequently (more than 1 out of 10 patients).
    d. Always.
    e. Other (please specify)
19. For which tumor sites do you perform patient specific LET or variable RBE calculations? [Select applicable responses]
    a. Base of skull
    b. Brain
    c. Breast
    d. Craniospinal irradiation
    e. Spine/paraspinal
    f. Head and neck
    g. Soft tissue abdomen (e.g., liver, pancreas)
    h. Lung
    i. Esophagus
    j. Prostate
    k. Other pelvis (e.g., sarcoma, gynecologic)
    l. Other sites (please specify)

20. Which RBE-related quantities do you calculate for clinical decision making? [Select applicable responses]
    a. LET distribution.
    b. Variable RBE distribution.
    c. Variable RBE-weighted dose distribution.
    d. Track-end distribution.
    e. NTCP with variable RBE.
    f. TCP with variable RBE.
    g. Other quantities (please specify)
21. Please specify the software/system you use to perform these calculations. [Select all applicable responses]
    a. RayStation
    b. Eclipse
    c. Monaco
    d. In-house software
    e. TOPAS/Geant4
    f. MCsquare
    g. Other (please specify)
22. Other Monte Carlo software (please specify)

VII. Consideration of variable RBE or LET retrospectively

23. Is a variable RBE or LET sometimes considered retrospectively when analyzing patient outcome at your institution?
    a. Yes
    b. No

CONDITIONAL:

IF YES: please answer following questions

ELSE: move on to next topic VIII

24. When do you consider variable RBE or LET retrospectively? [Select applicable responses]
    a. Routinely when comparing outcome with photon therapy.
    b. When analyzing clinical trials comparing proton and photon therapy.
    c. When trying to understand unexpected toxicity or recurrence for an individual proton therapy patient.
    d. Within a (retrospective) study to estimate parameters of a variable RBE model.
    e. In another way. (please specify)
25. For which tumor sites have you considered variable RBE retrospectively? [Select all applicable responses]
    a. Base of skull
    b. Brain
    c. Breast
    d. Craniospinal irradiation
    e. Spine/paraspinal
    f. Head and neck
    g. Soft tissue abdomen (e.g., liver, pancreas)
    h. Lung
    i. Esophagus
    j. Prostate
    k. Other pelvis (e.g., sarcoma, gynecologic)
    l. Other sites (please specify)

VIII. Wish list of needs regarding proton RBE

26. Do you feel the need for more education regarding clinical proton RBE?
    a. Yes
    b. No

CONDITIONAL:

IF YES: please answer the following question

ELSE: move on to next question Q28

27. What kind of education format would be relevant and suitable for you? [Select all applicable responses]
    a. Dedicated teaching articles or reviews
    b. Educational sessions at conferences
    c. Webinars
    d. Topic-specific workshops
    e. Other (please specify)

28. Do you think there is a need for more in vitro experiments to understand clinical proton RBE?
    a. Yes
    b. No

CONDITIONAL:

IF YES: please answer the following question

ELSE: move on to next question Q30

29. Which experiments would you propose?
    - Please specify

30. Do you think there is a need for more in vivo pre-clinical experiments to understand clinical proton RBE?
    a. Yes
    b. No

CONDITIONAL:

IF YES: please answer the following question

ELSE: move on to next question Q32

31. Which experiments would you propose?
    - Please specify

32. Do you think there is need for a multi-institutional database of patient outcomes focusing on the question of proton RBE?
    a. Yes
    b. No
